# Phylogeny of seven *Bulinus* species originating from endemic areas in three African countries, in relation to the human blood fluke *Schistosoma haematobium*

**DOI:** 10.1186/s12862-014-0271-3

**Published:** 2014-12-21

**Authors:** Rima Zein-Eddine, Félicité Flore Djuikwo-Teukeng, Mustafa Al-Jawhari, Bruno Senghor, Tine Huyse, Gilles Dreyfuss

**Affiliations:** Institute of Neuroepidemiology and Tropical Parasitology INSERM UMR 1094, Faculties of Medicine and Pharmacy, 2 Docteur Raymond Marcland Street, 87025 Limoges, France; Faculty of Health Sciences, University of Montagnes, P.B 208, Banganté, Cameroon; Laboratory of Microbiology, CNRS UMR 7276, Faculties of Medicine and Pharmacy, 2 Docteur Raymond Marcland Street, 87025 Limoges, France; Institute of Research for Development, URMITE, UM63, CNRS 7278, IRD 198, International Campus of Han, IRD B.P, 1386 Dakar, Senegal; Biology Department, Royal Museum for Central Africa, Leuvensesteenweg, 13, B-3080 Tervuren, Belgium

**Keywords:** *Bulinus* species, *Schistosoma haematobium*, Phylogeny, Genetic diversity, Host-parasite interactions

## Abstract

**Background:**

Snails species belonging to the genus *Bulinus* (Planorbidae) serve as intermediate host for flukes belonging to the genus *Schistosoma* (Digenea, Platyhelminthes). Despite its importance in the transmission of these parasites, the evolutionary history of this genus is still obscure. In the present study, we used the partial mitochondrial cytochrome oxidase subunit I (cox1) gene, and the nuclear ribosomal ITS, 18S and 28S genes to investigate the haplotype diversity and phylogeny of seven *Bulinus* species originating from three endemic countries in Africa (Cameroon, Senegal and Egypt).

**Results:**

The cox1 region showed much more variation than the ribosomal markers within *Bulinus* sequences. High levels of genetic diversity were detected at all loci in the seven studied species, with clear segregation between individuals and appearance of different haplotypes, even within same species from the same locality. Sequences clustered into two lineages; (A) groups *Bulinus truncatus, B. tropicus, B. globosus* and *B. umbilicatus*; while (B) groups *B. forskalii, B. senegalensis* and *B. camerunensis*. Interesting patterns emerge regarding schistosome susceptibility: *Bulinus* species with lower genetic diversity are predicted to have higher infection prevalence than those with greater diversity in host susceptibility.

**Conclusion:**

The results reported in this study are very important since a detailed understanding of the population genetic structure of *Bulinus* is essential to understand the epidemiology of many schistosome parasites.

## Background

Snails of the genus *Bulinus* (Müller, 1781) serve as intermediate hosts for larval development of the parasite species belonging to the *Schistosoma haematobium* species group in Africa, the Eastern Mediterranean and Madagascar [1], as for other Trematode species like paramphistomes. The *S. haematobium* blood flukes are responsible for 112 million infections in Africa with an incidence exceeding 50% in some communities [2]. The geographic distribution of these parasites is determined by the presence of their intermediate hosts [3]. Indeed, members of the *Bulinus* genus have an extensive distribution throughout Africa, some areas of the Middle East and the Mediterranean countries [4]. It is divided into four species groups: *B. africanus*, *B. forskalii*, *B. reticulatus* and the *B. truncatus/tropicus* complex [5]. Among the 37 species that compose these four groups, several are known or suspected to act as intermediate hosts for larval stages of the *Schistosoma* parasites*.* However, the role of each species in the transmission of the disease varies considerably between and within countries [4]. For example, in Cameroon *B. truncatus* is more implicated than *B. globosus* in the transmission of urinary schistosomiasis [6], while this is not the case in Senegal, where *B. globosus, B. senegalensis* and *B. umbilicatus* are known as the main intermediate hosts for *S. haematobium* [7].

The interpretation of these specific snail-parasite relationships requires a deeper understanding of the past events that have affected the population diversity and distribution of *Bulinus* species. Furthermore, such information is essential for assessing the geographical distribution of variation in genes associated with resistance in bulinid snails. Previous studies showed that the above four *Bulinus* species groups were stable throughout the majority of phylogenetic analyses and demonstrated the monophyletic status of these groups with both variable and highly conserved genes [8-10]. However, these studies were restricted to the basic phylogenetic level and no phylogeographic studies including estimates of haplotype and nucleotide diversity were performed.

In this investigation, we used a suite of population genetic and phylogenetic analyses to characterize populations of seven *Bulinus* species collected from endemic areas in three African countries (Cameroon, Senegal and Egypt), using one mitochondrial gene (the protein coding cytochrome oxidase subunit I (cox1) gene and three nuclear genes (18S rRNA, 28S rRNA, and the Internal Transcribed Spacer (ITS)). We tried to explain their present diversity in relation with schistosomiasis transmission.

## Results

### Population genetic analysis

#### Mitochondrial genome diversity

A data matrix of 1031 bp was used in this analysis (644 bp from the Folmer region and 387 bp from the Asmit region). All mtDNA sequences were found to be highly variable. Within species, the global haplotype diversity was very high in our samples ranging from 0.85 in *B. umbilicatus* to 0.99 for *B. senegalensis*. Furthermore, our estimate of nucleotide diversity ranged from 0.016 in *B. camerunensis* population to 0.052 for *B. senegalensis* (Table 1).

**Table 1:** **Sequence diversity in mitochondrial and nuclear ribosomal genes from seven** ***Bulinus*** **species collected in different African countries**

	**Countries**	**n**	***u***	***Hd***	***π***	***D (P-value)***	***Fs (P-value)***
**Cox1**							
*B. truncatus*	**All**	**56**	**35**	**0.911**	**0.050**	−1.834*	−6.209*
	Cameroon	24	20	0.955	0.042	−1.712	−0.249
	Egypt	20	4	0.508	0.023	0.844	1.997
	Senegal	12	9	0.961	0.053	−0.867	−4.159
*B. globosus*	Cameroon	20	18	0.978	0.035	−1.082	−1.351
*B. senegalensis*	**All**	**50**	**45**	**0.992**	**0.052**	**−1.611**	**−8.626**
	Cameroon	16	14	0.964	0.051	−0.364	−0.141
	Senegal	34	30	0.985	0.053	−1.666	−2.707
*B. tropicus*	Cameroon	10	8	0.932	0.045	−0.271	−1.531
*B. forskalii*	Cameroon	10	8	0.933	0.051	−0.027	−2.667
*B. umbilicatus*	Senegal	8	5	0.850	0.035	1.466	2.921
*B. camerunensis*	Cameroun	6	4	0.899	0.016	−0.8374	1.655
**18S**							
*B. truncatus*	**All**	**30**	**3**	**0.157**	**0.001**	−0.483	−4.121
	Cameroon	10	1	0.000	0.000	-	-
	Egypt	10	1	0.000	0.000	-	-
	Senegal	10	3	0.378	0.002	−0.507	2.199
*B. globosus*	Cameroon	10	3	0.417	0.001	−1.233	−0.189
*B. senegalensis*	**All**	**20**	**9**	**0.863**	**0.002**	**−0.009**	**−4.792**
	Cameroon	10	7	0.853	0.002	0.168	−4.538
	Senegal	10	6	0.801	0.001	0.592	−0.658
*B. forskalii*	Cameroon	10	4	0.458	0.001	−0.612	0.172
**28S**							
*B. truncatus*	**All**	**30**	**13**	**0.505**	**0.007**	−1.956*	**−4.095**
	Cameroon	10	5	0.539	0.008	−1.116	−0.062
	Egypt	10	4	0.504	0.006	1.193	1.716
	Senegal	10	6	0.568	0.009	−1.323	−1.285
*B. globosus*	Cameroon	10	7	0.831	0.004	1.168	−0.946
*B. senegalensis*	**All**	**20**	**14**	**0.955**	**0.012**	**−0.159**	**−1.962**
	Cameroon	10	8	0.893	0.011	−1.375	−1.785
	Senegal	10	9	0.956	0.013	1.168	−0.946
*B. forskalii*	Cameroon	10	7	0.834	0.012	1.365	7.499**

Note.— *n*: number of individuals sequenced, *U*: number of unique haplotypes within countries, *Hd*: haplotype diversity, *π*: nucleotide diversity, *D*: Tajima’s *D* statistic, *Fs*: Fu’s *Fs* statistic.

*P < 0.05 ; **P < 0.0.

Within countries, as expected haplotype diversity is lower but exceeds 0.800 in the three countries (Table 1). The number of *B. truncatus* unique haplotypes found in the Northern region of Africa (Egypt) is lower than that found in the Sub-Saharan Africa (Cameroon and Senegal). There is also higher nucleotide diversity found within *B. truncatus* populations sampled from Cameroon and Senegal compared to Egypt.

#### Ribosomal genome diversity

##### Ribosomal RNA (18S)

A data matrix of 852 bp was used in this analysis and the sequences were found to be highly similar. The highest haplotypes diversity was again observed within *B. senegalensis* species (Hd = 0.86), while the lowest one was observed within *B. truncatus* (Hd = 0.157), confirming the results obtained from the cox1 data (Table 1).

##### Ribosomal RNA (28S)

A data matrix of 855 bp was used in this analysis, the sequences were found to be more variable than 18S, and therefore higher values of haplotype and nucleotide diversity were observed (Table 1). Haplotype diversity exceeds 0.5 in all species, but again the highest Hd was detected in *B. senegalensis* (Hd = 0.95).

##### Ribosomal RNA (ITS)

ITS data was only available for six species from 17 different localities. Therefore, these data were only analyzed at a basic phylogenetic level and not at the population level. ITS sequences had an extreme amount of length variation, with a range of 600 to 990 bp. The shortest ITS lengths were noted in *B. globosus*, thus indicating probable deletion events. The largest ITS sequences were found in *B. tropicus* species. The sequences were found to be highly variable.

### Genetic divergence between and within species

The diversification index (*Fst*) between the seven studied species was calculated using the combined data matrix (Table 2). The largest divergence was observed between *B. forskalii* and *B. umbilicatus* populations (*Fst* = 0.903), whereas those of *B. truncatus* and *B. tropicus* are the most closely related (*Fst* = 0.206), as expected since these two species are part of the same group.

**Table 2:** **Estimation of fixation index (** ***Fst*** **) over sequence pairs from different** ***Bulinus*** **species using the combined cox1, 18S, and 28S data matrix**

**Species**	***B. truncatus***	***B. tropicus***	***B. senegalensis***	***B. camerunensis***	***B. forskalii***	***B. globosus***
***B. tropicus***	0.206					
***B. senegalensis***	0.538	0.600				
***B. camerunensis***	0.622	0.699	0.777			
***B. forskalii***	0.618	0.703	0.761	0.623		
***B. globosus***	0.523	0.599	0.721	0.791	0.793	
***B. umbilicatus***	0.659	0.711	0.817	0.899	0.903	0.774

The average within species diversity using the combined data matrix ranged from 0.005 to 0.040 (Table 3). The most heterogeneous species were *B. senegalensis* and *B. forskalii*, while individuals from *B. umbilicatus* are the most homogeneous, but this might be the result of the small simple size of the latter species.

**Table 3:** **Estimation of average evolutionary divergence over sequence pairs within species using the combined cox1, 18S and 28S data matrix**

**Species**	**Cox1-Folmer**	**Cox1-Asmit**	**18S**	**28S**	**Combined data matrix**
***B. truncatus***	0.007 ± 0.002	0.093 ± 0.009	0.002 ± 0.001	0.009 ± 0.001	0.026 ± 0.001
***B. tropicus***	0.020 ± 0.003	0.005 ± 0.009	0.002 ± 0.002	0.076 ± 0.008	0.024 ± 0.002
***B. senegalensis***	0.087 ± 0.006	0.049 ± 0.006	0.002 ± 0.001	0.011 ± 0.002	0.038 ± 0.002
***B. forskalii***	0.046 ± 0.006	0.052 ± 0.007	0.000 ± 0.000	0.122 ± 0.008	0.040 ± 0.003
***B. globosus***	0.055 ± 0.006	0.065 ± 0.008	0.001 ± 0.000	0.014 ± 0.003	0.024 ± 0.002
***B. umbilicatus***	0.002 ± 0.002	0.070 ± 0.011	0.002 ± 0.002	0.004 ± 0.002	0.005 ± 0.001
***B. camerunensis***	0.002 ± 0.002	0.022 ± 0.007	-	-	-
**Over all mean distance**	0.117 ± 0.009	0.134 ± 0.009	0.005 ± 0.002	0.038 ± 0.003	0.076 ± 0.003

### Demographic history

DnaSP v5.10.1 was used to perform Tajima’s and Fu’s neutrality tests in order to detect violations of mutation-drift equilibrium caused by selection or changes in population size. Within *B. truncatus*, the two tests were significantly negative using cox1 sequences, while no significant results were obtained when samples from individual countries were analyzed, perhaps in part because of the smaller sample sizes. Tajima’s *D* was significantly negative in the 28S dataset*.* Although these two statistics were originally introduced as tests of neutrality, Fu’s test is considered a powerful test to detect past population expansion. Therefore, significant negative values, such as those seen here, can indicate population expansion especially in Cameroonian and Senegalese *B. truncatus* populations. Within other species, the estimates of the two statistics were not significant in all regions, which indicate neutral evolution for these species in the studied countries.

### Phylogeny reconstruction

**Figure 1:**
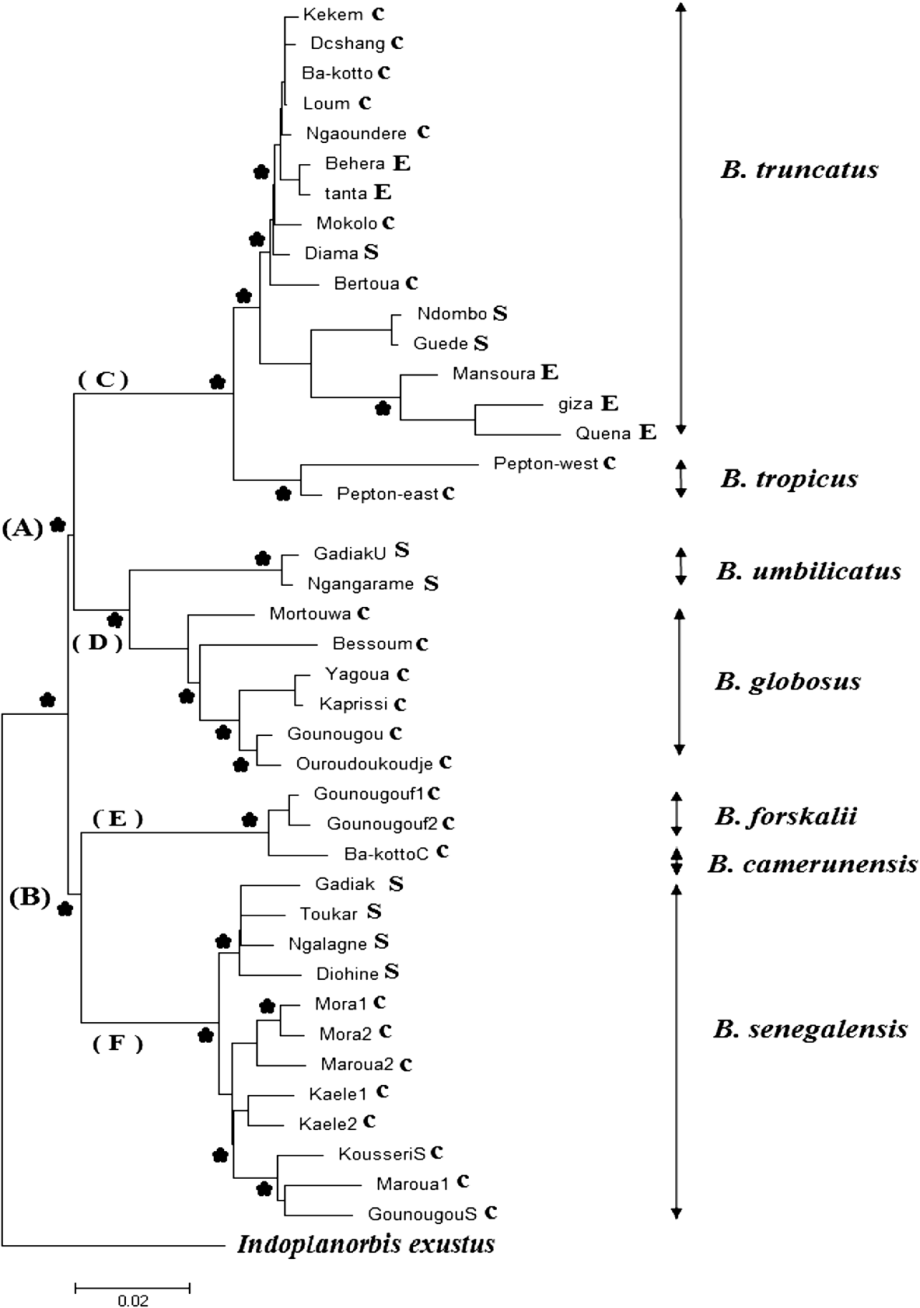
**Detailed view of the seven** ***Bulinus*** **species within the maximum-likelihood estimates (1000 bootstrap replicates) using combined data matrix (All genes together with TVM + G as selected model).** An asterisk indicates node support ≥ 80 for all methods used (parsimony, distance, and likelihood bootstrap analyses), with *Indoplanorbis exustus* as outgroup. Scale bar indicates 2 substitutions per 100 sites. Each terminal branch in the tree is marked using a letter country code (C: Cameroon, E: Egypt, and S: Senegal). Each internal branch represent monophyletic groups.

The phylogenetic trees produced for all gene fragments together and for each gene separately were very similar, as were the threes obtained using three different tree-building algorithms (maximum likelihood (ML), maximum parsimony (MP) and neighbor-joining (NJ)). All analyses confirm the monophyletic status of the *Bulinus* genus (bootstrap support of 93.3%; see Figure 1).

#### Combined data matrix

The results from all genes were combined together for 40 *Bulinus* specimens, as this improved resolution and resulted in higher bootstrap values for internal nodes compared to the single gene trees. The three different tree-building algorithms were largely congruent (Figure 1). All seven species were supported by high bootstrap values and split into two clusters: cluster A comprised members of the *B. truncatus/tropicus* complex and the *B. africanus* group, while cluster B represented the *B. forskalii* group*.*

The cluster A is split into two subclades (bootstrap value of 94%), the first subclade C contains sequences that are restricted to either *B. truncatus* or *B. tropicus,* with Cameroonian *B. truncatus* haplotypes branching off earlier than those from Senegal and Egypt. This could indicate that these *B. truncatus* haplotypes were introduced into Egypt from Sub-Saharan countries. The second subclade D includes *B. globosus* and *B. umbilicatus* haplotypes.

As for the second cluster B, the haplotypes split again into two subclades supported by bootstrap values of 99%. Subclade E includes sequences from *B. forskalii* and *B. camerunensi*s, while subclade F contains sequences that are restricted to *B. senegalensis*, which is itself split into two groups: the first with Cameroonian haplotypes and the second with Senegalese haplotypes (Figure 1).

#### mtDNA (Cox1 Folmer and Asmit regions)

**Figure 2:**
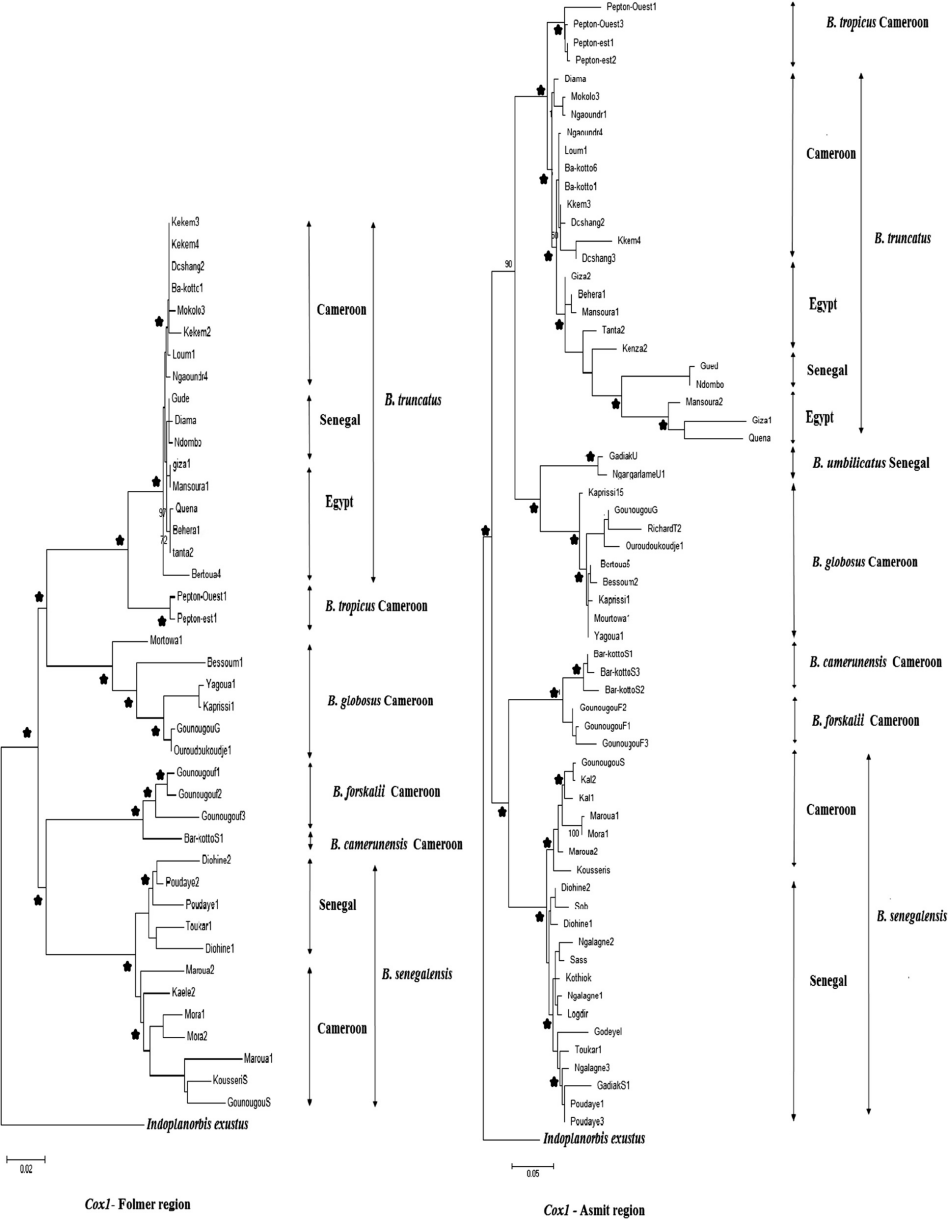
**Detailed view of the seven** ***Bulinus*** **species within the maximum-likelihood estimates (1000 bootstrap replicates) using cox1 Folmer and Asmit region (All genes together with GTR + G as selected model).** An asterisk indicates node support ≥ 80 for all methods used (parsimony, distance, and likelihood bootstrap analyses), with *Indoplanorbis exustus* as outgroup. Scale bar indicates 2 substitutions per 100 sites for Cox Folmer region and 5 substitutions per 100 sites for Cox Asmit region.

The cox1 region carried a strong phylogenetic signal, with clear haplotype clustering and high bootstrap support, although with higher bootstrap values for the Folmer region (Figure 2). The phylogenetic analysis indicated the same clustering as found with the combined data matrix. However, due to the higher number of cox1 sequences individuals could be grouped according to the country of origin. For example three clear *B. truncatus* clusters could be identified using both Folmer and Asmit regions, with again Cameroonian *B. truncatus* haplotypes branching off earlier than those from Senegal and Egypt (Figure 2). In addition, the *B. senegalensis* haplotypes split into two lineages, the first originated from Cameroon and the second from Senegal, the clustering within Senegal was however less supported (Figure 2).

#### Nuclear rRNA (18S, 28S and ITS)

**Figure 3:**
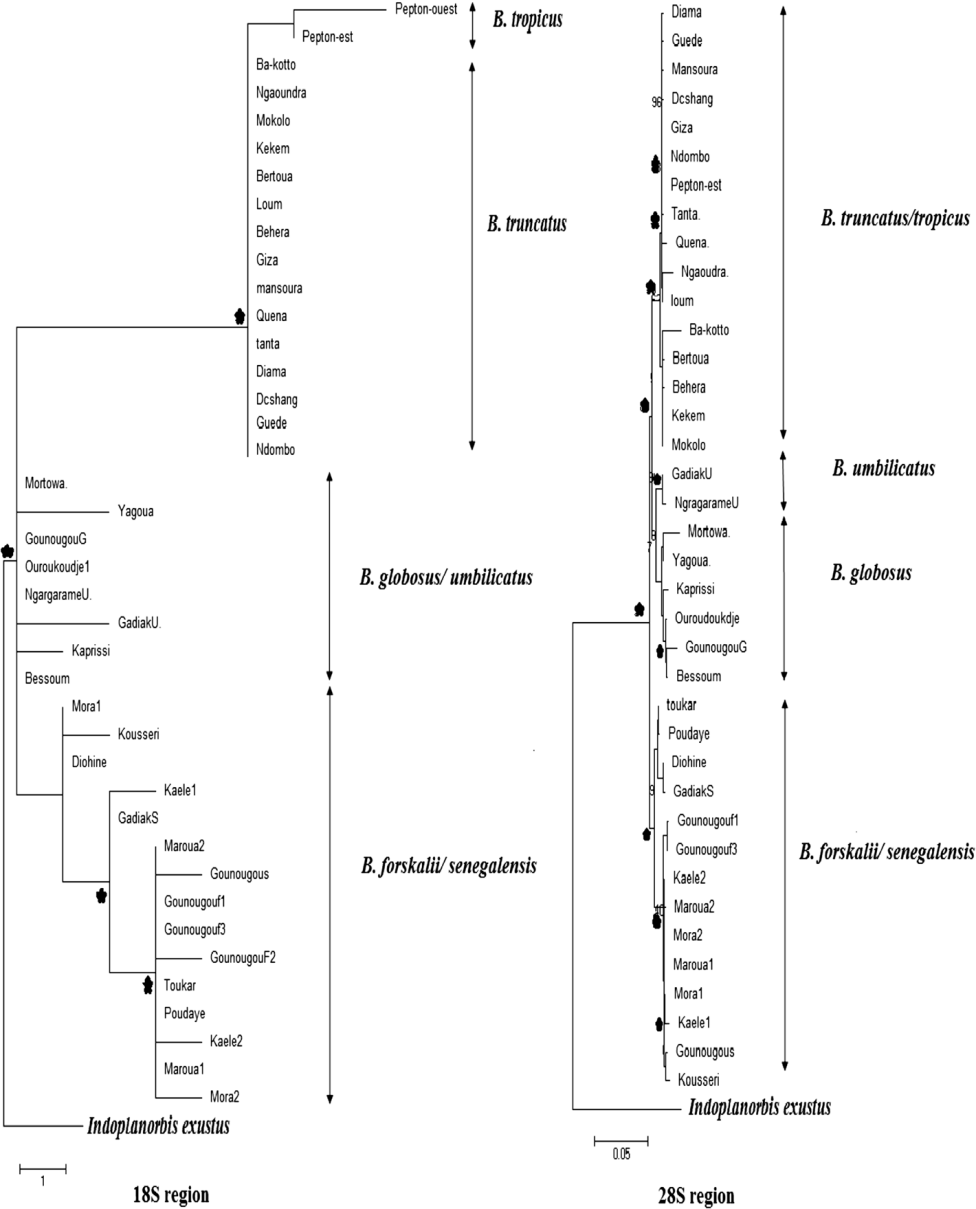
**Detailed view of the seven** ***Bulinus*** **species within the maximum-likelihood estimates (1000 bootstrap replicates) using 18S and 28S (All genes together with GTR + G as selected model).** An asterisk indicates node support ≥ 80 for all methods used (parsimony, distance, and likelihood bootstrap analyses), with *Indoplanorbis exustus* as outgroup. Scale bar indicates 5 substitutions per 100 sites for 28S region.

The phylogenetic trees generated using the nuclear rRNA (18S, 28S and ITS) confirm the major clades described by both the combined data matrix and the cox1 region (Figure 3). However, the 18S region was not able to classify the sequences according to their species. This is the case of the *B. africanus* group where there was no clear segregation between *B. globosus* and *B. umbilicatus*. Similarly, the three species within the *B. forskalii* group, *B. senegalensis, B. camerunensis* and *B. forskalii* all grouped together (Figure 3).

## Discussion

### Population genetic analysis

The mtDNA sequences showed high haplotype and nucleotide diversity, with a clear segregation according to species and locality. Many different haplotypes were found, with approximately one out of three to four snail specimens having a different haplotype within the same locality. This high level of genetic diversity was higher than that found by Nalugwa et al. [11] in *B. truncatus* and *B. forskalii* (from Cameroon) where one snail out of six to seven individuals had different mtDNA haplotypes using the same fragment [11].

Of the nuclear fragments, 18S was the least variable, while 28S showed an intermediate level of nucleotide variation compared to the ITS fragment which was the most variable. These results are comparable to that found in the work of Jørgensen et al., [8] where they also observed a lower variation in 18S compared to 28S in 26 *Bulinus* taxa representing the four species groups currently recognized in this genus from different African countries [8]. Despite this intermediate nucleotide diversity in 28S, the haplotype diversity was high and comparable to that found with the cox1 region.

This high genetic diversity detected at all loci, both within and between species is probably influenced by several factors:

i) Firstly, geographical isolation is probably the most dominant factor to explain the global diversity within species from different countries. In fact, *Bulinus* populations investigated in the present study originated from geographically distant countries, limiting gene flow between them. This reproductive isolation might have allowed independent evolution and divergence of these populations leading to distinct lineages. Indeed, our results showed that *B. truncatus* and *B. senegalensis* populations from the same country clustered together and formed different lineages (Figure 2).
ii) Secondly, *Bulinus* species are hermaphrodite and can reproduce both by cross-fertilization and/or self-fertilization [12], and each of these modalities induces different genetic consequences [13]. *B. globosus* and *B. senegalensis* are mainly outcrossing and therefore they showed high levels of genetic variability.
iii) Thirdly, despite the fact that *B. truncatus* is preferentially self-fertilizing, it is a tetraploid species [14], and studies have shown that diversification is high in polyploid species [15], which might explain this relatively high haplotype diversity detected within this snail species.

### Snail genetic diversity and host parasite compatibility

The relationship between *Schistosoma* and *Bulinus* species is very specific and varies in according to geographic location [7,16]. For schistosome - snail interactions, empirical data suggest local adaptation of parasites which result in higher infection levels of local snail populations [17]. This in turn could affect the genetic diversity of these snails from one infection site to another [17].

In Cameroon, our results showed lower levels of genetic diversity for *B. truncatus* than for *B. globosus* and *B. senegalensis* at all loci. In fact, according to Schmid-Hempel [15], and Coltman et al., [19], a loss of genetic variation in host population can increase susceptibility to parasitism [18,19], basing on this statement higher infection and transmission rates should be expected in those *B. truncatus* populations. Indeed, experimental infections of *B. globosus* and *B. truncatus* from Cameroon with different isolates of local miracidia were carried out to study snail-parasite compatibility [6]. Their findings suggest that *B. truncatus* might be more implicated than *B. globosus* in *S. haematobium* transmission.

On the other hand, *B. forskalii* is ubiquitous in Cameroon and it is often present in association with *B. senegalensis*. It acts as intermediate host for *S. guineensis* [20]*.* The results reported in the present study revealed very high levels of genetic diversity in *B. forskalii* at all loci, which is in accordance with the work of Gow et al. [20], who have detected high level of microsatellite polymorphism and gene diversity within *B. forskalii* populations in Cameroon. Furthermore, according to Tchuem Tchuenté et al. [1], there is a decrease or cessation in *S. guineensis* transmission in some major foci in Cameroon, indicating a progressive extinction for this parasite species in this country [20]. This parasite extinction is most likely the result of hybridization between *S. guineensis* and *S. haematobium*, but also is maybe influenced by the high levels of genetic diversity within Cameroonian *B. forskalii* populations since a gain of genetic variation can decrease susceptibility to parasitism [19].

In Egypt, only *B. truncatus* acts as intermediate host for urinary schistosomiasis. *B. truncatus* snails are recorded in water bodies throughout Egypt. It is most abundant in large canals, and decreases in density as the water approaches and flows into drains [21]. In this country, urinary schistosomiasis is prevalent along the Nile Valley from the Delta region to Upper Egypt. A seasonal analysis showed that *B. truncatus* snails are year-round infected with schistosomes [22]. In the present study, we have noted a relatively high genetic diversity within Egyptian *B. truncatus* populations*.* In parallel, according to El-Khoby 2000, there is a decrease in the prevalence of *S. haematobium* in Egypt during the last years, and *S. mansoni* has almost totally replaced *S. haematobium* in Lower Egypt and is spreading into Upper Egypt [23]. Furthermore, the haplotypes detected from the Nile Delta were different from those found in Upper Egypt. Prevalence of *S. haematobium* in Upper Egypt sites was higher than that found in the Nile Delta [23]. This might be explained by a variety of factors that are probably related to a lower socioeconomic status of people who live in rural sites (Upper Egypt) and are more employed in agriculture so that they are more exposed to infection. Another factor might be related to higher genetic diversity within populations of *B. truncatus* in Nile Delta.

In Senegal, our results showed higher diversity in *B. truncatus* populations than those in Egypt and Cameroon. This might be linked to the fact that Senegalese *B. truncatus* populations are implicated in the transmission of *S. bovis* and *S. haematobium* x *S. bovis* hybrids, but not of pure *S. haematobium* [24], suggesting a strong role for parasite genetics in influencing the diversity of their intermediate hosts.

### Phylogeny of bulinids from Cameroon, Egypt and Senegal in relation to schistosomiasis

Schistosomiasis transmission depends on the active role of the intermediate host. The disease is thus closely related to rural development of water resources and snail population expansion [25]. Therefore, we cannot talk about parasite distribution without referring to the distribution of its intermediate host. Our results showed lower diversity within *B. truncatus* populations originating from North Africa (Egypt) compared to those originating from Sub-Saharan Africa (Cameroon and Senegal) at all loci. Many factors can explain this lower diversity in Egypt such as selection, drift or probably a relatively recent introduction since specimens from Cameroon and Senegal are basal to them in lineage C (Figures 1 and 2), which may suggest that *B. truncatus* may have colonized Egypt from Sub-Saharan Africa. In addition, a previous hypothesis suggested that schistosomiasis has appeared earlier in various parts of sub-Saharan Africa and then became widely distributed in North Africa during prehistoric wet phases [26]. In fact, the physical and human environment of the Nile valley and delta provided increasingly favorable conditions for the transmission and spread of schistosomiasis caused by *S. haematobium* after the development of irrigation agriculture during the early Pharaonic period. Although the snail fossil record is poor in the ancient Nile valley and delta, *B. truncatus* host snails were probably widespread, also indicated by their recovery from irrigated areas and wells elsewhere in the Middle East.

## Conclusion

Molecular studies provide opportunities to investigate the contribution of snail genetics to variation in disease burden and pathology. The results reported in this study are very important since a detailed understanding of *Bulinus* population’s genetic variation has become increasingly relevant to the transmission of many schistosome parasites. A novel insight from our study is the high levels of genetic diversity detected within *Bulinus* populations originating from different African countries, which might affect the susceptibility of *Bulinus* species to parasite infection. In addition, the highest genetic diversity was detected within populations of *B. forskalii* and *B. senegalensis*. Furthermore, our results suggest that *B. truncatus* may have colonized Egypt from Sub-Saharan Africa. Further studies are required with higher simple size on a large scale to understand the demographic histories of these snails’ populations.

## Methods

### Taxon sampling and DNA extraction

Snails of the *Bulinus* genus were sampled from Cameroon, Egypt and Senegal. The climate is of Mediterranean type in Egypt, tropical or equatorial in Cameroon, and mainly tropical in Senegal. This allowed the discovery of as much genetic variability as possible even within the same species from different countries. Table 4 summarizes the main characteristics of the investigated sites. Seven different species have been studied: *B. camerunensis, B. forskalii, B. globosus, B. senegalensis, B. tropicus, B. truncatus* and *B. umbilicatus*. The taxonomic identity of the sampled snails was initially determined based on shell morphology using the field identification key of Kristensen (1987). Snails were collected from their habitats and conserved in 70% ethanol until genetic analyses.

**Table 4:** **Location and sampling information for the seven** ***Bulinus*** **species collected from 45 sites in Cameroon, Senegal and Egypt**

**Species**	**Localities**	**GPS Co-ordinate**	**Pre in % references**	***N***
*B. truncatus*	Bertoua, Cameroon	04°35′20″ N, 13°40′52″ E	43.3	4
	Kékem, Cameroon	05°09′47″ N, 10°00′37″ E	1 - 4	4
	Mokolo, Cameroon	10°44′00″ N, 13°46′04″ E	25 - 49	2
	Barombi-kotto, Cameroon	04°28′04″ N, 09°15′02″ E	68.9	4
	Dschang, Cameroon	05°26′43″ N, 10°04′01″ E	1 - 4	4
	Ngaoundéré, Cameroon	07°18′59″ N, 13°35′22″ E	1 - 4	4
	Loum, Cameroon	04°42′58″ N, 09°44′11″ E	62.8	2
	Behera, Egypt	30°33′28″ N, 30°42′14″ E	1.8	4
	Kafer-zayat, Egypt	30°48′08″ N, 30°48′06″ E	0.45	2
	Tanta, Egypt	30°45′32″ N, 31°00′07″ E	0.45	2
	Kenesa West, Egypt	30°09′39″ N, 31°10′09″ E	0.26	2
	Giza, Egypt	30°08′29″ N, 31°04′37″ E	0.26	4
	Mansouria, Egypt	30°00′28″ N, 31°12′07″ E	0.26	4
	Quena, Egypt	26°10′23″ N, 32°09′58″ E	4.78	2
	Diama, Senegal	15°26′10″ N, 16°14′02″ E	-	4
	Guedé, Senegal	16°31′52″ N, 14°47′33″ E	-	4
	Ndmobo, Senegal	16°26′08″ N, 15°41′52″ E	-	4
*B. tropicus*	Petponoun-East, Cameroon	05°37′59″ N, 10°38′09″ E	95	5
	Petponoun-West, Cameroon	05°37′59″ N, 10°38′07″ E	95	5
*B. globosus*	Mourtouwa, Cameroon	10°12′42″ N, 14°11′28″ E	50-100	4
	Yagoua, Cameroon	10°20′58″ N, 15°13′55″ E	25-49	2
	Gounougou, Cameroon	09°04′33″ N, 13°42′25″ E	78.8	2
	Ouroudoukoudje, Cameroon	09°05′53″ N, 13°43′22″ E	57.3)	2
	Djalingo, Cameroon	10°23′53″ N, 15°15′11″ E	-	4
	Bessoum, Cameroon	09°07′00″ N, 13°15′11″ E	77.2	6
	Richard Toll, Senegal	16°27′40″ N, 15°41′15″ E	-	4
*B. umbilicatus*	Gadiak, Senegal	14°51′49″ N, 16°03′24″ E	66.97	4
	Ngangarame, Senegal	14°34′60″ N, 16°28′60″ E	60.50	4
*B. senegalensis*	Maroua, Cameroon	10°34′56″ N, 14°19′39″ E	-	4
	Mora, Cameroon	11°03′03″ N, 14°08′58″ E	-	4
	Gounougou, Cameroon	09°04′33″ N, 13°42′25″ E	78.8	2
	Kaélé, Cameroon	10°06′07″ N, 14°27′03″ E	>50	4
	Kousseri, Cameroon	12°04′59″ N, 15°01′60″ E	-	2
	Toukar, Senegal	14°31′51″ N, 16°28′36″ E	61.31	4
	Diohine, Senegal	14°29′48″ N, 16°30′17″ E	72.54	4
	Gadiak, Senegal	14°51′49″ N, 16°03′24″ E	66.97	4
	Poudaye, Senegal	14°48′48″ N, 16°37′13″ E	62.50	6
	Logdir, Senegal	14°30′48″ N, 16°30′13″ E	48.53	2
	Ngalagne, Senegal	14°24′29″ N, 16°47′43″ E	91.53	6
	Kotior, Senegal	14°28′32″ N, 16°32′13″ E	43.33	2
	Godiyel, Senegal	13°41′00″ N, 13°26′00″ E	53.03	2
	Sob, Senegal	14°29′20″ N, 16°26′58″ E	34,55	2
	Sass, Senegal	14°30′03″ N, 16°24′04″ E	45,61	2
*B. camerunensis*	Barombi-kotto, Cameroon	04°28′04″ N, 09°15′02″ E	68.9	6
*B. forskalii*	Gounougou, Cameroon	09°04′33″ N, 13°42′25″ E	78.8	10
Total				164

Note. N: sample size, Pre: prevalence rate of human infection with *S. haematobium.*

Genomic DNA was extracted from snails using the QiAamp DNA Mini Kit (Qiagen®, France) following manufacturer’s instructions. A Nanodrop ND-1000 spectrophotometer was used to quantify and check DNA purity.

### DNA amplification and sequencing

Four markers were selected for sequencing: the protein coding cytochrome c oxidase subunit I (cox1), and three nuclear ribosomal genes (ITS, 18S and 28S). Partial sequences from the four genes were obtained after PCR amplification. Various combinations of the used primers are presented in Table 5.

**Table 5:** **Primers used for PCR amplification and sequencing**

**Primer name**	**Primer sequence**	**Forward or reverse**	**Source**
Cytochrome oxidase subunit 1			
CO1 (LCO490)	5’-GGT CAA CAA ATC ATA AAG ATA TTG G-3’	Forward	Folmer et al., [27]
CO2 (HCO2198)	5’-TAA ACT TCA GGG TGA CCA AAA AAT CA-3’	Reverse	
Asmit 1 (ATI)	5’-TTT TTT GGG CAT CCT GAG GTT TAT-3’	Forward	Bowles et al., [28]
Asmit 2 (AT2)	5’-TAA AGA AAG AAC ATA ATG AAA ATG-3’	Reverse	
Internal transcribed spacer of the ribosomal gene complex			
ETTS1	5’- TGC TTA AGT TCA GCG GGT -3’	Reverse	Kane and Rollinson, [29]
ETTS2	5’- TAA CAA GGT TTC CGT AGG TGA A -3’	Forward	
Small Subunit rRNA (SSU)			
18SBaso3F	5’-GTG CTC TTC NCT GAG GGT CC-3’	Forward	Jørgensen et al., 2011 [8]
18SBaso9R	5’-TAC GGA AGC CTT GTT ACG A-3’	Reverse	
Large Subunit rRNA (LSU)			
Baso2600F	5’-GGA ATC CGA CTG TCT AAT TAA AAC-3’	Forward	Jørgensen et al., 2011 [8]
Baso3600R	5’-CRG ATG GAT GGT AGC YTC GCA CC-3’	Reverse	

For cox1 and ITS, 25 μL PCR mixtures were set up with 0.5 U Taq polymerase (Qiagen), 5 μL 10x buffer, 2–4 μL MgCl2 (depending on template and primers), 0.8 μL dNTPs, 0.5 μL from each primer (20 ng/μL). For 18S and 28S rRNA; amplification was performed with High Fidelity Taq DNA polymerase (Roche) in a 50 μL total reaction volume with standard reaction conditions since this enzyme is designed to increase yield and fidelity when amplifying longer fragments. A standard concentration of 500 ng/μL was used as template for all PCR reactions.

The PCR conditions varied for each primer combination. Cycling conditions for both cox1 and ITS were as follows: preheat step at 95°C for 5 min followed by 35 cycles of denaturation at 94°C for 10 sec, annealing at 40°C for 30 sec and extension at 72°C for 1 min; final extension step at 72°C for 10 min. Amplification for both 18S and 28S involved i) a preheat step at 94°C for 2 min, ii) 10 cycles, each including denaturation at 94°C for 15 sec, annealing at 43°C for 30 sec and extension at 72°C for 45 sec, iii) 15 cycles including the same conditions except the extension step which was expanded to 5 sec per cycle, iv) a final extension step at 72°C for 10 min.

PCR products were purified using QIAquick PCR Purification Kit (Qiagen®, France), then sequenced using an ABI Prism BigDye® Terminator v1.1 Cycle Sequencing Ready Reaction Kit (PE, Applied Biosystems) following manufacturer’s instructions, and run on an ABI 377 automated sequencer.

### Data analyses

The obtained sequences were used to perform BLAST searches [30] via the National Center for Biotechnology Information (NCBI) GenBank to assure that the sequences matched the sequenced genes. Sequence chromatograms were checked using SeqScape v2.5. Nucleotide sequences were multiple aligned using CLUSTAL X [31], and submitted to GenBank database under the accession numbers [GenBank: KJ135287-KJ13508 and KJ157326-KJ157508].

#### Population genetic analysis

DnaSP v5.10.1 [32] was used to estimate global and regional levels of haplotype and nucleotide diversity for the seven species, and to conduct Tajima’s and Fu’s neutrality tests for patterns on non-neutral evolution within each species. We also used DnaSP to estimate the diversification index *(Fst)* between each pair of species.

MEGA v5 [33] was used to estimate the average evolutionary divergence over sequence pairs within species for each gene. The optimal model of sequence evolution that best fits the data was evaluated using FindModel: for cox1-Folmer region (GTR + G), Cox Asmit-region (GTR + G), 18S (GTR + I), 28S (K81 + G) and combined data matrix (GTR + I + G). Standard error estimates were obtained by a bootstrap procedure (500 replicates).

#### Phylogenetic analyses

In the case of interpopulation diversity, data matrices were constructed (with unique haplotypes) for individual genes separately, and then for all combined genes. All phylogenetic analyses were carried out using MEGA. Evolutionary relationships between the haplotypes were inferred using maximum parsimony (MP), maximum likelihood (ML), and Neighbor-joining (NJ) methods. The different types of analyses were subjected to 1000 bootstrap replicates as a means for testing the reliability of individual branches within the generated tree. *Indoplanorbis exustus* (Deshayes, 1834) (Planorbidae: Bulininae) was selected as outgroup in analyses of cox1 (Folmer and Asmit regions), 18S and 28S, as Morgan et al., [34] had placed this genus as sister group to *Bulinus* [34]*.* Trees using ITS data matrix were constructed with *Biomphalaria helophila* as outgroup since no ITS sequence of *I. exustus* was available. Parsimony inference was performed via a heuristic search using 1000 replicates of random sequence entry, tree-bisection-reconnection (TBR) branch swapping. Maximum likelihood and NJ trees were performed with the inferred substitution models as mentioned above.

### Availability of supporting data

The data sets supporting the results of this article are available in the Dryad.org repository, doi:10.5061/dryad.hf63h.

## References

[1] Tchuem Tchuenté LA, Southgate VR, Jourdane J, Webster BL, Vercruysse J (2003). *Schistosoma intercalatum*: an endangered species in Cameroon?. Trends Parasitol.

[2] Van der Werf MJ, Bosompem KM, Vlas SJ (2003). Schistosomiasis control in Ghana: case management and means for diagnosis and treatment within the health system. Trans R Soc Trop Med Hyg.

[3] Agatsuma T (2003). Origin and evolution of *Schistosoma japonicum*. Parasitol Int.

[4] Rollinson D, Kaukas A, Johnston D, Simpson A, Tanaka M (1997). Some molecular insights into schistosome evolution. Int J Parasito.

[5] Brown DS: **Freshwater snails of Africa and their medical importance.** In *CRC Press*. *Volume 2*. 2nd edition. Edited by Taylor and Françis ltd. London; 1994:208–247.

[6] Njiokou F, Teukeng E, Bilong Bilong CF, Njiné T, Same Ekobo A (2004). Experimental study of the compatibility between *Schistosoma haematobium* and two species of Bulinus in Cameroon. Bull Société Pathol Exot.

[7] Sène M, Southgate VR, Vercruysse J (2003). *Bulinus truncatus*, intermediate host of *Schistosoma haematobium* in the Senegal River Basin (SRB). Bull Société Pathol Exot.

[8] Jørgensen A, Madsen H, Nalugwa A, Nyakaana S, Rollinson D, Stothard JR, Kristensen TK (2011). A molecular phylogenetic analysis of *Bulinus* (Gastropoda: Planorbidae) with conserved nuclear genes. Zool Scr.

[9] Kane RA, Stothard JR, Emery AM, Rollinson D (2008). Molecular characterization of freshwater snails in the genus *Bulinus*: a role for barcodes?. Parasit Vectors.

[10] Jørgensen A, Kristensen TK, Madsen H (2008). A molecular phylogeny of apple snails (Gastropoda, Caenogastropoda, Ampullariidae) with an emphasis on African species. Zool Scr.

[11] Nalugwa A, Jørgensen A, Nyakaana S, Kristensen TK (2010). Molecular phylogeny of *Bulinus* (Gastropoda: Planorbidae) reveals the presence of three species complexes in the Albertine Rift freshwater bodies. Int J Genet Mol Biol.

[12] Jarne P (1993). Resistance genes at the population level. Parasitol Today Pers Ed.

[13] Jarne P, Charlesworth D: **Hermes meets Aphrodite: an animal perspective**. *Trends Ecol Evol* 1996, **24:**35–39. 10.1016/0169-5347(96)81085-8 21237773

[14] Brown DS, Shaw KM (1989). Freshwater snails of the *Bulinus truncatus/tropicus* complex in Kenya: tetraploid species. J Mollus Stud.

[15] Schmid-hempel S (1998). Parasites and flower choice of bumblebees. Anim Behav.

[16] Vera C, Mouchet F, Bremond P, Sidiki A, Sellin E, Sellin B (1992). Natural infection of Bulinus senegalensis by *Schistosoma haematobium* in a temporary pool focus in Niger: characterization by cercarial emergence patterns. Trans R Soc Trop Med Hyg.

[17] Minchella D, LoVerde PT: **Laboratory comparison of the relative success of*****Biophalaria glabrata*****stoks wiich are susceptible and insusceptible to infection with*****Schistosoma*****mansoni**. *Parasitology* 1993, **19:**335–344. 10.1017/s0031182000050502 6856336

[18] King KC, Lively CM (2012). Does genetic diversity limit disease spread in natural host populations?. Heredity.

[19] Coltman DW, Smith JA, Bancroft DR, Pilkington J, MacColl AD, Clutton-Brock TH, Pemberton JM (1999). Density-dependent variation in lifetime breeding success and natural and sexual selection in Soay rams. Am Nat.

[20] Gow JL, Noble LR, Rollinson D, Mimpfoundi R, Jones CS (2004). Breeding system and demography shape population genetic structure across ecological and climatic zones in the African freshwater snail, Bulinus forskalii (Gastropoda, Pulmonata), intermediate host for schistosomes. Mol Ecol.

[21] Dazo BC, Hairston NG, Dawood IK (1996). The ecology of *Bulinus truncatus* and Biomphalaria alexandrina and its implications for the control of bilharziasis in the Egypt-49 project area. Bull World Health Organ.

[22] Abdel-Nasser A (2008). Prevalence of urinary schistosomiasis and Infections with Trematode larval stages in *Bulinus truncatus* Snails from Qena Upper Egypt. J Appl Sci Res.

[23] El-Khoby T, Galal N, Fenwick A, Baraka R, El-Hawey A, Nooman Z, Habib M, Abdel-Wahab F, Gabr NS, Hammam HM, Hussein MH, Mikhail NN, Cline BL, Strickland GT (2000). The epidemiology of schistosomiasis in Egypt: summary findings in nine governorates. Am J Trop Med Hyg.

[24] Huyse T, Webster BL, Geldof S, Stothard JR, Diaw OT, Polman K, Rollinson D (2009). Bidirectional introgressive hybridization between a cattle and human schistosome species. PLoS Pathog.

[25] Kloos H, Thompson K (1979). Schistosomiasis in Africa: an ecological perspective. J Trop Geogr.

[26] David R: **The paleoepidemiology of schistosomiasis in Ancient Egypt.** *Soc Hum Eco Soc Humen Ecology* 2000, **9:**14.

[27] Folmer O, Black M, Hoeh W, Lutz R, Vrijenhoek R (1994). DNA primers for amplification of mitochondrial cytochrome c oxidase subunit I from diverse metazoan invertebrates. Mol Mar Biol Biotechnol.

[28] Bowles J, Blair D, McManus DP (1992). Genetic variants within the genus Echinococcus identified by mitochondrial DNA sequencing. Mol Biochem Parasitol.

[29] Kane RA, Rollinson D (1994). Repetitive sequences in the ribosomal DNA internal transcribed spacer of Schistosoma haematobium, Schistosoma intercalatum and Schistosoma mattheei. Mol Biochem Parasitol.

[30] Altschul SF, Gish W, Miller W, Myers EW, Lipman DJ (1990). Basic local alignment search tool. J Mol Biol.

[31] Thompson JD, Gibson TJ, Plewniak F, Jeanmougin F, Higgins DG (1997). The CLUSTAL_X windows interface: flexible strategies for multiple sequence alignment aided by quality analysis tools. Nuc Acids Res.

[32] Librado P, Rozas J (2009). DnaSP v5: a software for comprehensive analysis of DNA polymorphism data. Bioinforma Oxf Engl.

[33] Tamura K, Nei M, Kumar S (2004). Prospects for inferring very large phylogenies by using the neighbor-joining method. Proc Natl Acad Sci U S A.

[34] Morgan JAT, DeJong RJ, Jung Y, Khallaayoune K, Kock S, Mkoji GM, Loker ES (2002). A phylogeny of planorbid snails, with implications for the evolution of *Schistosoma* parasites. Mol Phylogenet Evol.

